# Stress drives premature hive exiting behavior that leads to death in young honey bee (*Apis mellifera*) workers

**DOI:** 10.1186/s40659-024-00569-z

**Published:** 2024-11-27

**Authors:** Jordan Twombly Ellis, Juliana Rangel

**Affiliations:** Department of Entomology, Texas A&M University, 2475 TAMU, College Station, TX 77843-2475 USA

**Keywords:** Accelerated age polyethism, Hypopharyngeal glands, Precocious foraging, *Varroa destructor*

## Abstract

**Background:**

The Western honey bee, *Apis mellifera*, is an economically important pollinator, as well as a tractable species for studying the behavioral intricacies of eusociality. Honey bees are currently being challenged by multiple biotic and environmental stressors, many of which act concomitantly to affect colony health and productivity. For instance, developmental stress can lead workers to become precocious foragers and to leave the hive prematurely. Precocious foragers have decreased flight time and lower foraging efficiency, which can ultimately lower colony productivity and even lead to colony collapse.

**Materials and methods:**

In this study, we tested the hypothesis that stress during pupal development can cause young workers to exit the hive prematurely before they are physically able to fly. This premature exiting behavior results in death outside the hive soon thereafter. To determine how various stressors may lead bees to perform this behavior, we subjected workers during the last pupal stage to either cold stress (26 °C for 24 h), heat stress (39 °C for 24 h), or *Varroa destructor* mite parasitization, and compared the rate of premature hive exits between stressed bees and their respective control counterparts. Upon emergence, we individually tagged focal bees in all treatment groups and introduced them to a common observation hive. We then followed tagged bees over time and monitored their survivorship, as well as their likelihood of performing the premature hive exiting behavior. We also dissected the hypopharyngeal glands of all treatment and control bees sampled.

**Results:**

We found that significantly more bees in all three treatment groups exited the hive prematurely compared to their control counterparts. Bees in all treatment groups also had significantly smaller hypopharyngeal glands than control bees.

**Conclusions:**

Our results suggest that premature hive exiting behavior is driven by stress and is potentially a form of accelerated age polyethism that leads to premature death.

**Supplementary Information:**

The online version contains supplementary material available at 10.1186/s40659-024-00569-z.

## Introduction

Honey bees (*Apis mellifera*) are the world’s most economically important pollinators, contributing over $209 billion annually to the world’s economy [1]. In the United States, managed honey bees provide over $15 billion in economic value every year by pollinating approximately one third of the agricultural crops included in an average person’s diet [2]. These values underscore the reason why their continued survival and prosperity is crucial for food security [3]. But despite their importance, the population of managed honey bees has been declining in the last few decades, with most beekeeping operations sustaining summer and winter losses that are much higher than those that are deemed acceptable [4, 5]. Annual colony losses can be attributed to several concomitant stressors, including parasites, pathogens, poor nutrition, pesticide exposure, and climate change, which can overwhelm colonies and often lead them to collapse [5–8]. In particular, extreme temperatures can cause decreased survivorship, foraging efficiency, and learning in workers [9, 10]. Similarly, bees stressed during development by the parasitic mite *Varroa destructor* also show decreased lifespans, lower foraging efficiency, and impaired learning capabilities [11, 12]. One behavioral response caused by developmental exposure to such stressors is accelerated age polyethism [13, 14].

Age polyethism is the ordered and predictable progression of task specializations that are undertaken by individual workers in eusocial insect colonies as they age [15, 16]. In honey bees, this age-related division of labor begins with recently emerged workers (one to three days of age) performing cell cleaning tasks. They then perform nursing tasks from day three until around day ten. After that, the bees start moving toward the periphery of the hive to perform activities such as processing food or guarding the colony [17, 18]. Finally, around day 21, they transition into foragers [19]. Foraging is the last task performed by adult workers, as a majority of them ultimately die in the field due to old age [20]. Workers do not typically exit the colony before they reach the foraging age, at which point they begin to take orientation flights for a few days to learn the local landmarks before venturing into flying long distances to procure food [17].

While the timeline of age polyethism in honey bees is well established, it can be flexible depending on a colony’s condition. For example, foragers can inhibit young bees from transitioning to perform foraging tasks in colonies with a high proportion of foragers [21]. This is achieved by foragers transferring ethyl oleate (released from their honey crop) to young bees via trophallaxis. Conversely, when a colony lacks enough older bees, there are physiological and social cues that can cause younger bees to prematurely take on roles that are typically done by older workers [18, 22]. This is termed accelerated age polyethism. One form of accelerated age polyethism is precocious foraging, which occurs when bees that are younger than the typical foraging age of 21 days (usually aged between days 8 and 10 post emergence) leave the colony to begin their foraging career [23]. Precocious foraging by young bees is a behavior that can be caused either by the loss of generations of foragers, stress, or both [24, 25]. Various types of stress experienced during development can cause precocious foraging, including *Varroa* mite parasitization and extreme temperatures [14, 26]. Precocious foraging may provide a temporary relief to colony needs; however, young workers generally have poor orientation and communication skills, and exhibit accelerated mortality when they forage prematurely [27, 28]. This can create a negative feedback loop in which the colony’s population of foragers is lost too quickly. In fact, mathematical models have demonstrated that high levels of precocious foraging can ultimately lead to colony collapse [28].

Previous studies have elucidated a few of the physiological and genetic mechanisms underlying accelerated age polyethism and precocious foraging behavior in honey bees [23, 29–31]. For example, one of the physiological features of workers that forage precociously is decreased hypopharyngeal gland (HPG) size [19]. HPGs are found in the bees’ heads and are largest in nurse-aged bees (i.e., those that are between the ages of six to ten days old), and smallest in forager bees (i.e., those that are 21 days old or older). The enlarged HPGs of typical nurse-aged bees are used to produce brood food and royal jelly, which are fed to developing workers and queens, respectively [32, 33]. As such, HPG size can be used as a marker of behavioral maturation because nurse bees have large HPGs and forager bees have small HPGs [34, 35]. Other physiological markers of accelerated age polyethism include increased juvenile hormone (JH) levels and decreased vitellogenin (Vg) levels [29].

Preliminary observations in our laboratory suggested the existence of an extreme example of premature hive exiting behavior performed by young bees: we observed very young workers that had been parasitized by *Varroa* mites during pupation walking out of the hive before they could fly. This led to them to die outside the hive shortly thereafter. This behavior of bees prematurely exiting the hive and dying outside the hive’s entrance will hereafter be referred to as “premature hive exiting behavior.” During our preliminary observations, the behavior was performed by workers between two and seven days of age who walked out of the hive until they dropped to the ground, given that they were unable to fly. The bees would then walk away until they died. Studies have documented the existence of an altruistic self-removal of foraging-aged bees [36, 37]. However, there is no prior research explaining a hive exiting behavior observed in young bees that are unable to fly. This led us to ask the question: what are the factors that drive young workers to perform this premature hive exiting behavior that ultimately leads to their untimely death?

We hypothesized a few factors that might be causing premature hive exiting behavior in honey bees. Because we first observed it as a response to heavy *Varroa* mite parasitization, we first hypothesized that the behavior could be a way for the colony to limit parasite or disease spread. Another hypothesis was that this could be a form of extremely accelerated age polyethism driven by severe stress, whereby very young bees attempt to fly prematurely, but instead of flying out, they die upon exiting the hive because of their youth and inability to fly. If this were the case, and the behavior was a form of extremely accelerated behavioral maturation, we further hypothesized that bees that exited the hive prematurely would have smaller HPGs compared to their age-matched control counterparts. This would support the hypothesis that premature hive exiting occurs in bees that resemble older bees in some physiological markers, but are unable to fly because of their young age and therefore, perish before they are able to perform any foraging tasks. We found no behavioral evidence to indicate that any prematurely exiting bees are attempting to fly and/or forage. However, our results suggest that one potential physiological marker of this behavior (i.e., small HPGs in prematurely exiting bees) resembles the physiology of bees that undergo accelerated aging.

## Materials and methods

### Subjecting workers to developmental stress

We sourced all colonies for this study from the Janice and John G. Thomas Honey Bee Facility located on the RELLIS Campus of Texas A&M University in Bryan, TX. To explore the drivers of premature hive exiting behavior in young workers, we conducted trials in the summers of 2020 and 2021 by stressing developing bees during the last stage of pupation with either a cold stress treatment, a heat stress treatment, or *Varroa* mite parasitization. Bees that were stressed by any of those treatments had independent control cohorts of age-matched untreated bees sourced from the same colony.

To subject developing bees to temperature stress, we sourced frames of emerging workers from healthy colonies. We used at least two frames per colony, one for the treatment group, and one for the untreated control group. Once the frames were identified, we brushed off all adult bees and placed the frames in five-frame “nucleus” hives in two separate incubators. For the cold stress trials, we placed one frame in an incubator kept at 26 °C and a corresponding control frame from the same colony in an incubator kept at a normal brood temperature of 35 °C [15]. For the heat stress trials, we placed one frame in an incubator kept at 39 °C and a corresponding control frame from the same colony in another incubator kept at 35 °C. All frames were left in the incubators for 24 h, and we removed all the bees that emerged from their cells during that period. We then placed the two nucleus hives back in their respective incubator. After a 24 h, we collected all bees that emerged from the frames. This process allowed us to stress the bees for exactly 24 h (not less) during the last day of pupation, ensuring that all the bees that we tagged as treatment or control bees were the same age. All emerged bees from each frame were collected and labeled on their thorax with a numbered tag of a distinct color so that each bee had an identity associated with her respective treatment [38]. Once all the bees were tagged, we placed them in a cage with a honey feeder and put the cage back in the 35 °C incubator until the evening, at which point the bees were introduced into a three-frame observation colony. Each glass-walled observation colony consisted of two frames of brood, one frame of food, and approximately 6000 workers [39]. Bees in the observation hives, along with their respective queens, were sourced from existing colonies within the apiary.

For the *Varroa* mite stress trials, frames containing pupating bees that were stressed by mite parasitization were sourced from a colony with a 4% mite infestation or higher on the adult bee population, which is considered a high mite load [40]. The mite load was determined by performing an alcohol wash on the colony in the week prior to sourcing the bees. As a worker emerged from pupation, we examined her and her cell to determine whether she had been parasitized by *Varroa* during development. Based on that observation, we classified each emerging adult as being in either the mite-stressed treatment group, or the non-parasitized control group. Bees were not used in the trial if they had visible deformities, such as crinkled wings, an extended proboscis, or the inability to walk properly.

We weighed focal bees before tagging them in four of the cold stress trials and two of the mite parasitization trials to determine whether weight could play a role in the premature hive exiting of bees. We did not take weight data from bees during the heat stress experiment because we found no difference in the aforementioned trials and did not see it as necessary. We once again labeled the bees in each cohort with a uniquely colored number tag, put them into a small honey-coated cage, and placed them in the 35 °C incubator until dusk, which is when bees typically stop foraging. This ensured that the worker population in the observation colony was high. To introduce tagged bees into the hive as seamlessly as possible, we sprayed the bees with sugar water and placed their cage atop the observation hive on the top corner opposite the side of the entrance hole. The high worker population and the long distance from the introduction point to the hive’s entrance made it less likely for hive bees to reject and drag the introduced bees out of the colony. The sugar water also enticed the hive bees to groom the introduced bees, helping their chemical profiles to blend within hours, and increasing the acceptance rate of foreign bees into the observation hive [38]. We introduced bees into four different foster observation colonies to control for the effect of the observation hive on the performance of the premature hive exiting behavior. Lastly, we engineered a trap outside each observation hive consisting of a large plastic tub with petroleum jelly coating the inner sides. This helped us trap all bees that exited the hive prematurely and were unable to fly, while allowing any foragers that accidentally fell into the tub to fly away without issue.

### Behavioral analysis

In each trial, we monitored the acceptance of focal bees into the colony, as well as their survivorship once they were introduced. Acceptance was checked 12 h after introduction and a bee was considered to be accepted if she was present in the colony the morning after it was introduced. Once all the tagged bees were accepted into their respective observation hives, we checked for the presence of labeled bees twice a day for seven consecutive days. We did this by scan-sampling both sides of the observation hive and reading the numbers and colors of the tags on each bee into a digital voice recorder, as done previously [41]. Those data were collected to verify that the treatment bees were indeed developmentally stressed, as decreased survivorship is usually indicative of stress caused by *Varroa* parasitization or extreme temperatures [9, 11]. The data were later transcribed onto an Excel spreadsheet. Bees that were recorded to be present in the hive the day after they were introduced were considered as accepted, while bees that were never seen post-introduction were considered as having been rejected by the foster colony. We also took video recordings of the outside entrance of the hive for 30 min per day to record the behavior live as it happened. However, our main method of analysis consisted in us checking the trap outside the hive for the presence of prematurely exiting bees every hour during daylight hours from day one to day seven post-introduction. For the premature hive exiting assessment, we only counted bees that had been accepted into the colony, recording the identity (numbered tag), date, and time when bees prematurely exited the hive. We collected only live bees from the trap, as this allowed us to know with certainty that they had exited on their own, as opposed to them having died in the hive and having been dragged out by undertaker bees. Our study was conducted using observation hives with an entrance that was located one meter off the ground. It is important to note that in most apiary and wild settings, hives are generally located a few to several feet off the ground [42], and therefore, a bee that has dropped to the ground and cannot fly would generally be unable to return to the hive on her own.

### Hypopharyngeal gland (HPG) dissections

We measured the average diameter (µm) of ten HPG acini from bees across all treatment groups, as well as of bees from all relevant control groups, to compare their average HPG size to that of young bees that prematurely exited the hive. For each collected bee that performed the premature hive exiting behavior, we collected four other control bees. The first two were tagged bees that were introduced into the observation hive on the same day as the prematurely exiting bees. These control bees were collected by opening a sliding door engineered into the brood nest area, spraying the bees with vanilla water to keep them from flying away, and picking two randomly chosen tagged bees from the nest. First, we took one tagged bee from the same treatment group as the prematurely exiting bee. Then, we took a tagged bee from the corresponding control group. The next two controls were forager-aged bees that were also tagged and introduced to the hive on the same day as the prematurely exiting bees. However, we waited approximately ten days after the end of the trial to collect those forager controls so that they would have enough time to reach the foraging age. We collected those bees by standing outside the entrance of the observation hive and catching tagged bees that were returning to the hive. Because the first two control bees were the same age as the prematurely exiting bees, we hypothesized that significant differences in average acinus size between bees in the treatment and control groups (of the same age) could suggest that HPG size plays a role in the likelihood of bees prematurely exiting the hive. We further hypothesized that prematurely exiting bees would have similarly sized HPGs to their foraging control counterparts. If HPG size does not play a role in this behavior, we would expect foragers to have smaller HPGs than young prematurely exiting bees, as foragers naturally have smaller HPGs than young bees in undisturbed colonies [43].

We stored all the treatment and control bees at − 80 °C and later dissected their HPGs following the protocol outlined by Corby-Harris and Snyder [44]. In brief, we removed the head, removed the faceplate, and pipetted 20 μL of a phosphate buffer solution to enable the glands to float to the top of the head. Once removed from the head and placed on a microscope slide, 20 μL of Giemsa stain was used to stain the glands blue. The glands were then examined for size using a compound microscope. We used a Moticam Connect microscope camera to measure the diameter of 10 acini per HPG. We then calculated the average acinus size per bee across all treatment groups. We measured the average acinus size for at least twelve bees per treatment group, across all groups.

### Statistical analysis

We used Wilcoxon rank-sum tests to discern differences in bee weight across treatment groups. We constructed survivorship curves using a Kaplan–Meier estimator and contrasted the curves using log-rank tests. We analyzed differences in the rate of premature hive exiting behavior of bees across treatment groups using a Chi-square (*X*^*2*^) test. If this behavior was a form of extremely accelerated age polyethism where no foraging takes place (as we hypothesized), we would expect similar correlations between all stressors. We determined differences in the average HPG acinus size between treatment groups by performing Kruskal–Wallis tests followed by pairwise Dunn’s tests. We used the statistical program R for all data analysis and used α = 0.05 as the threshold of statistical significance for all tests.

## Results

We performed a total of 17 trials in the summers of 2020 and 2021 (Table 1). We sourced focal bees from different colonies for all temperature stress trials. We conducted five trials for the cold stress experiment, with a combined sample size for those trials being 639 cold-stressed bees and 522 control bees. Likewise, we performed five trials for the heat stress experiment, with a combined sample size for those trials of 617 heat-stressed bees and 580 control bees. For both the cold and heat stress treatments, we introduced focal bees into two different foster observation colonies to control for any potential effect caused by the foster colony. Finally, we conducted seven trials for the *Varroa* mite stress experiment and sourced the focal bees from different colonies that had varying mite loads above the treatment threshold. Our combined sample size for all the *Varroa*-related trials was 738 mite-stressed bees and 749 no-mite control bees.

**Table 1:** Number of honey bee workers that were introduced and monitored for premature hive exiting behavior in either the cold stress (26 °C for 24 h), heat stress (39 °C for 24 h), or *Varroa* mite parasitization experimental treatment groups

Experimental treatment group	*n*	Control treatment group	*n*
Cold stress (26 °C)	639	Normal broodnest temperature (35 °C)	522
Heat stress (39 °C)	617	Normal broodnest temperature (35 °C)	580
*Varroa* mite stress	738	No *Varroa* mite stress	749

Each experimental group of workers had an age-matched control group of workers that was monitored at the same time for comparison

We examined the acceptance rate between stressed and control bees to determine if undergoing any of the treatments led to low acceptance of tagged bees by observation colonies. We found that 95.8% of the cold-stressed bees and 97.5% of their respective controls were accepted by the foster observation colony (*X*^*2*^ test *p*-value = 0.107). Likewise, 92.3% of the heat-stressed bees and 96.2% of their respective controls were accepted by the foster observation colony (*X*^*2*^ test *p*-value = 0.168). Finally, 86.7% of the mite-stressed bees and 89.5% of no-mite controls were accepted (*X*^*2*^ test *p*-value = 0.242). We therefore concluded that our treatments did not affect the acceptance of focal individuals into the observation colonies used in the experiments. We also found no significant differences in weight between bees that exited the hive prematurely and those that did not in either the cold-stressed group (*p*-value = 0.620), or in the *Varroa* mite stressed group (*p*-value = 0.832; (Supplementary Fig. 1).

In terms of survivorship, adult bees that were exposed to 26 °C for 24 h during the last day of pupal development died significantly sooner than their control counterparts (*p*-value < 0.00001; Supplementary Fig. 2A). Likewise, bees that were exposed to 39 °C for 24 h during the last day of pupal development died significantly sooner than their control counterparts (*p*-value < 0.00001; Supplementary Fig. 2B). We observed a similar numerical trend of earlier mortality in bees that were parasitized by *Varroa* mites, but this difference compared to control bees was not significant (*p*-value = 0.062; Supplementary Fig. 2).

**Fig. 1:**
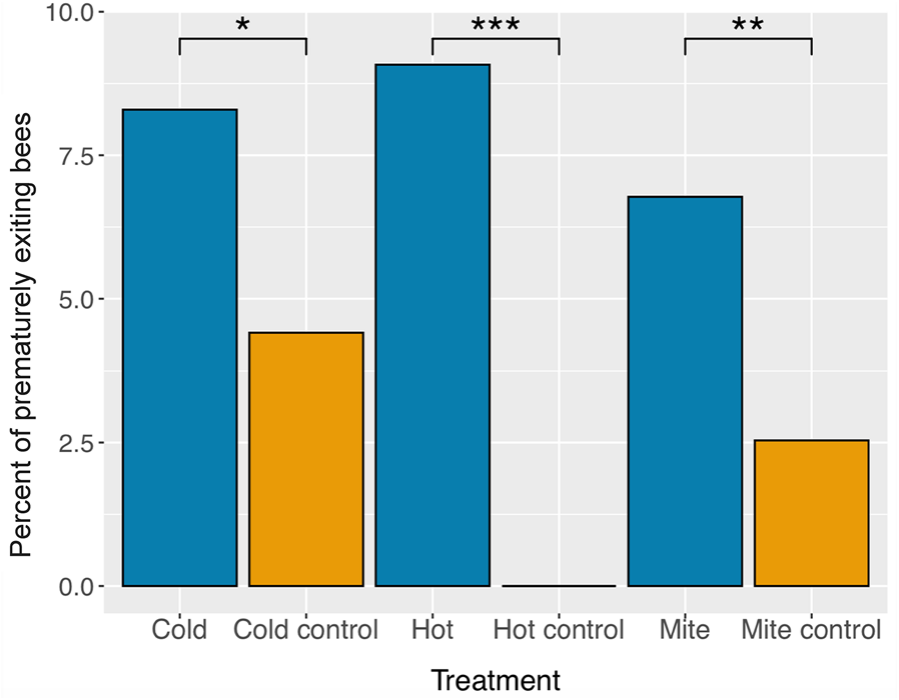
Comparisons of the percentage of honey bee workers that performed the premature hive exiting behavior across treatment groups (blue bars) and their respective control groups (orange bars). Differences between each treatment and control group were evaluated using a chi-squared test. A significantly higher percentage of bees prematurely exited the hive in the cold stress treatment group (*p* = 0.004), the heat stress treatment group (*p* < 0.0001), and the *Varroa* mite parasitization group (*p* < 0.0001), compared to their control counterparts. Asterisks above the brackets indicate significant pair-wise differences between the treatment and control groups (“**” for *p* < 0.01) and (“***” for *p* < 0.001). Sample sizes were as follows: cold stress group: 639; cold control group: 522; heat stress group: 617; heat control group: 580; mite stress group: 738; no mite group: 749

Our behavioral observations revealed that, once bees that prematurely exited were outside the hive and in the trap below, they would stand motionless or walk away slowly until they were collected. If bees were collected outside before death and placed back inside the hive, they would repeat the behavior. Furthermore, there was no evidence of them being taken out of the hive by undertaker bees. Video recordings of this behavior showed similar patterns in all the bees that performed this behavior (Supplementary Video 1). We found a significantly higher rate of premature hive exiting behavior in young bees that were exposed to the cold stress treatment compared to their respective age-matched controls (*X*^*2*^ = 2.66,* p*-value = 0.004; Fig. 1). Likewise, there was a significantly higher rate of premature hive exiting behavior in young bees that were exposed to the heat stress treatment compared to their age-matched controls (*X*^*2*^ = 6.71, *p*-value < 0.00001; Fig. 1). Finally, we found that bees that had been parasitized by *Varroa* mites performed the premature hive exiting behavior at a significantly higher rate than those that had not been parasitized (*X*^*2*^ = 3.89, *p*-value < 0.0001; Fig. 1).

**Fig. 2:**
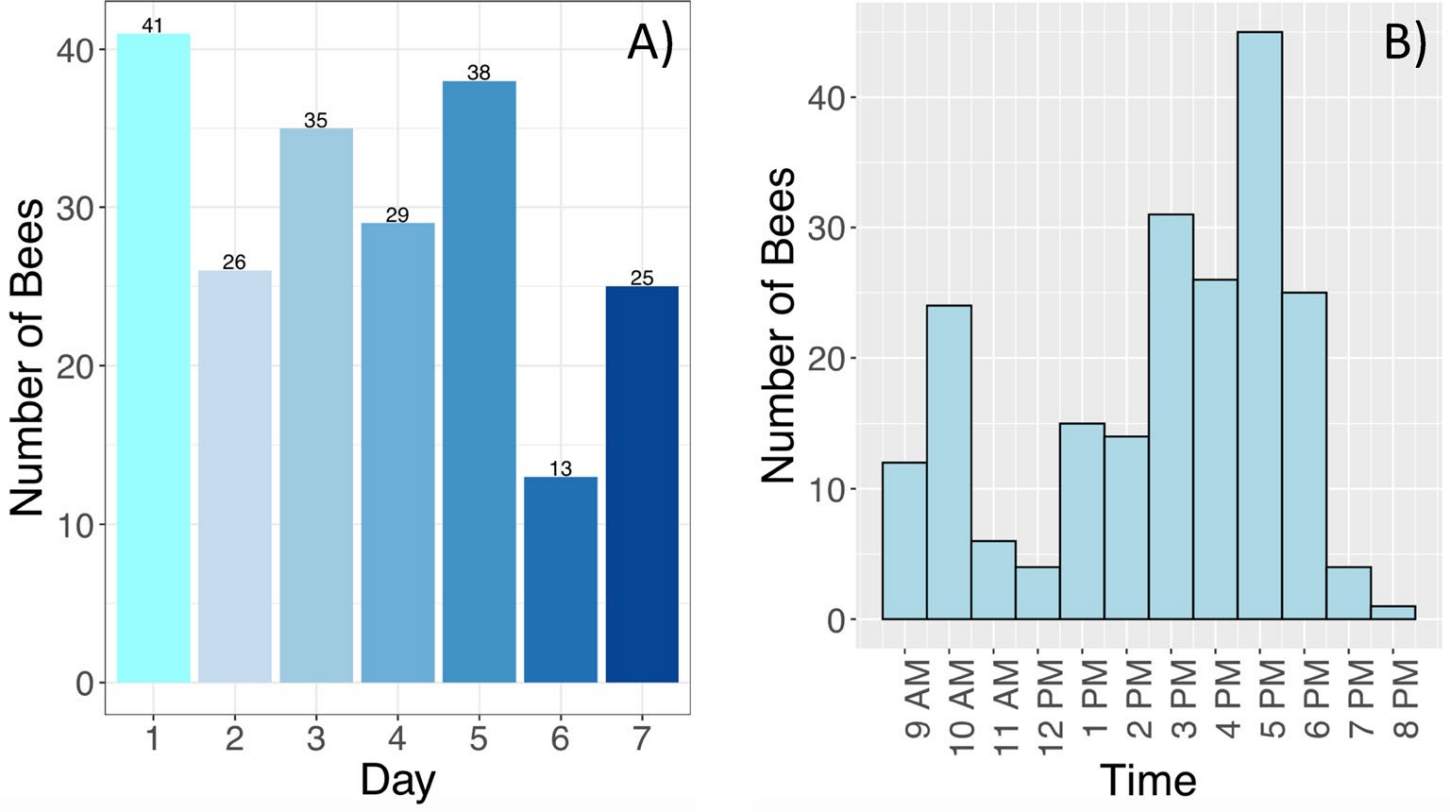
Day and time during which the premature hive exiting behavior was observed. **A** Shows the number of combined hive exiting occurrences per day, with the highest number of bees prematurely exiting the hive on day 1 of a trial, and the lowest number being on day 6 of a trial. **B** Shows the cumulative number of premature hive exiting occurrences observed based on the time of day, with a spike in premature hive exiting occurrences happening in the late afternoon, at around 5:00 p.m

Moreover, by recording the date and time of the occurrence of this behavior, we found that the performance of the behavior occurred across all seven days of observation, with an average of 30 bees performing it each day (Fig. 2A). There was a peak of performance on day 1 (n = 38 bees) and a trough on day 6 (n = 13 bees). The average age of bees that exited the hive prematurely was 4.66 days. We also found that bees that performed the behavior did so primarily in the late afternoon, with a peak performance (n = 45 bees) happening around 17:00 (Fig. 2B).

We found no significant difference in HPG acinus size between stressed bees and unstressed control bees in either the age-matched or forager control groups for all three stressors tested (Dunn’s tests. Cold stress trials: stressed vs. unstressed age-matched controls *p*-value = 0.885; stressed vs. unstressed forager controls *p*-value = 1.0. Heat stress trials: stressed vs. unstressed age-matched controls *p*-value = 1.000; stressed vs. unstressed forager controls *p*-value = 0.988. Mite stress trials: stressed vs. unstressed age-matched controls *p*-value = 1.0; stressed vs. unstressed forager controls *p*-value = 0.42). Therefore, for statistical purposes, for each experiment we combined the control groups into two groups: general age-matched controls, and forager-aged controls. The sample size for each group is shown in Table 2.

**Table 2:** Number of honey bee workers that were dissected for hypopharyngeal gland acinus size analysis

Treatment	Prematurely exiting bees (*n*)	Age-matched control bees (*n*)	Forager control bees (*n*)
Cold stress	25	50	25
Heat stress	22	29	12
*Varroa* mite stress	61	57	21

The sample size (*n*) is denoted for the prematurely hive exiting bees, the age-matched control bees, and the forager control bees that were sampled for each of the three treatment groups tested

**Fig. 3:**
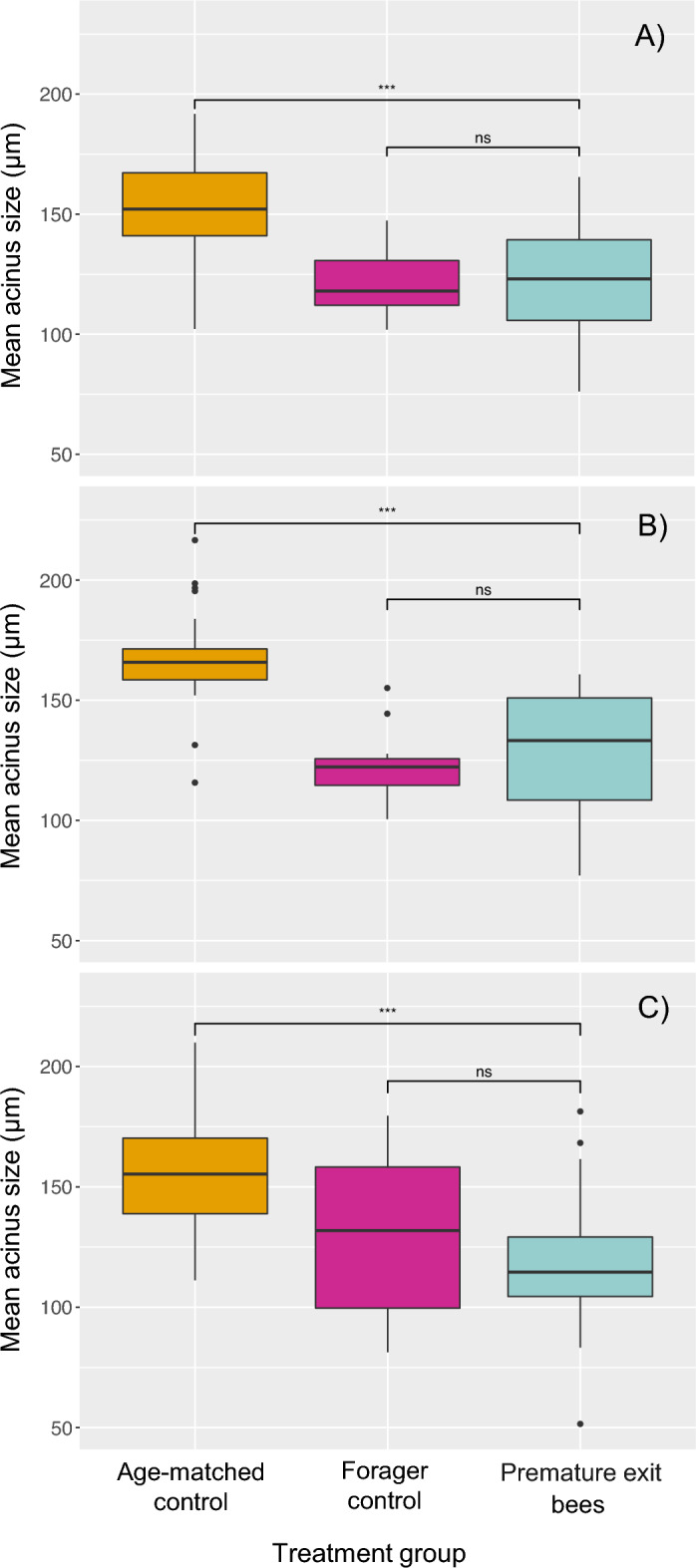
Median and distribution of mean hypopharyngeal gland (HPG) acinus size (µm) of workers that prematurely exited the hive as well as their age-matched and forager control counterparts. For all three panels, HPG acinus size is shown in orange for age-matched control bees, magenta for forager-aged control bees, and teal for prematurely exiting bees. Kruskal–Wallis tests were run to detect overall differences between means and Dunn’s tests were run for pairwise comparisons. **A** Mean HPG acinus size of bees in the cold stress treatment group (26 °C for 24 h) was significantly lower for prematurely exiting bees compared to their age-matched counterparts (*p* < 0.0001), but similarly sized compared to the forager controls (*p* = 0.588). **B** Mean acinus size for bees in the heat stress treatment group (39 °C for 24 h) was significantly lower for prematurely exiting bees compared to their age-matched counterparts (*p* < 0.00001), but similarly sized compared to the forager controls (*p* = 0.576). **C** Mean acinus size for bees in the *Varroa* mite parasitization group was significantly lower for prematurely exiting bees compared to their age-matched counterparts (*p* < 0.00001), but similarly sized compared to the forager controls (*p* = 0.135). Asterisks (“***”) above brackets represent statistically significant differences between groups (at *p* < 0.001), and “n.s.” indicates no significant differences between groups. Sample sizes: **A** age-matched control: 50, forager control: 25, premature exit: 25; **B** age-matched control: 29, forager control: 12, premature exit: 22; **C** age-matched control: 57, forager control: 21, premature exit: 61

Interestingly, we found that the mean HPG acinus size in bees sampled for the cold stress experiments was significantly different across treatment groups (Kruskal–Wallis, F_2,98_ = 33.67; *p*-value < 0.00001). Pairwise comparison using Dunn’s tests showed that bees that prematurely exited the hive from the cold stress treatment group had significantly smaller acini than their age-matched control counterparts *(p-*value < 0.00001), but similarly sized acini compared to their forager-age control counterparts (*p*-value = 0.588; Fig. 3A). Likewise, HPG acinus size in the heat stress trials differed significantly between treatment groups (Kruskal–Wallis, F_2,61_ = 35.31; *p*-value < 0.00001). Pairwise Dunn’s tests also revealed that the prematurely exiting bees from the heat stress treatment group had significantly smaller acini than their age-matched control counterparts (*p-*value < 0.00001), but had similarly sized acini compared to their older forager control counterparts (*p*-value = 0.576; Fig. 3B). Finally, HPG acinus size in the mite stress trials differed significantly between treatment and control groups (Kruskal–Wallis, F_2,137_ = 51.078; *p*-value < 0.00001). Pairwise Dunn’s tests found the same result as before: prematurely exiting bees from the mite parasitized group had significantly smaller acini than their age-matched control counterparts (*p-*value < 0.00001), but similarly sized acini compared to their older forager control counterparts (*p*-value = 0.135; Fig. 3C).

## Discussion

In this study, we report the existence of a premature hive exiting behavior that leads directly to death in very young workers that under typical colony conditions should perform tasks associated with the nurse stage in the age polyethism program for honey bees. Instead of performing nursing tasks, some of those bees exited the hive prematurely, dropped to the ground, were unable to fly, and died soon after. This premature exit was observed at higher rates in bees that were exposed to various types of stress during pupation: cold stress, heat stress, and *Varroa* mite parasitization. It was determined that this premature hive exiting behavior was not caused by the stress caused by bees being introduced to observation hives given that, across stressors, bees in all treatment groups had statistically similar acceptance rates as their control counterparts. This demonstrates that tagged individuals were not differentially removed by hive bees after their introduction. Our observational data further supported this conclusion. Furthermore, we found no significant differences in weight between prematurely exiting bees and the rest of the bees introduced into the observation colonies. Thus, we can infer that nutritional deficiencies did not seem to be a factor in the likelihood of bees performing the premature hive exiting behavior, although we did not test this hypothesis directly.

Our data also showed that the bees that were used as controls for the mite stress treatment suffered low survivorship compared to the control bees used in the cold or heat stress groups; this could have been because the no-mite control bees were sourced from the same colony as the mite-stressed bees, whereas the bees used in the temperature stress trials were sourced from different hives as their unstressed control counterparts. While this procedure was important in the mite stress trials to decrease genetic variability among bees in the treatment vs. control groups, it also introduced the likelihood that even non-parasitized brood exhibited some background levels of honey bee-associated viruses that are often present in colonies with high mite loads [45]. Previous work has shown that once a colony is infected with too many mites, the viruses they vector may be present even within non-parasitized bees due to oral transmission [46]. Despite this, we still observed significantly higher rates of premature hive exiting behavior in bees that were parasitized by mites, indicating that mite parasitization augments the likelihood of bees performing the behavior regardless of any background levels of viruses present in the hive.

Every stressor to which we subjected pupating bees caused stressed bees to exit the hive prematurely at significantly higher rates than their control counterparts. Those data support our hypothesis that this premature hive exiting behavior is not necessarily a disease-driven social immune response, but rather, it is driven by generalized developmental stress. Interestingly, the peak time during which the behavior was performed each day was around 5 p.m., which corresponds to the time at which bees perform orientation flights. However, our behavioral evidence does not indicate that the focal bees were attempting to either orient or forage. Instead, they dropped out of the hive and had no ability to return even in the absence of our plastic trap. For these reasons, we contend that these bees exited the hive before they could fly and subsequently died. Furthermore, we never observed physical contact between the bees that prematurely exited the hive and their nestmates prior to exiting. Therefore, more studies would be needed to attribute teleology to orient or forage to any prematurely exiting bees.

One physiological marker that is commonly associated with precocious foraging behavior is decreased HPG size. Hypopharyngeal glands secrete royal jelly, which is fed to developing larvae [47]. Nursing behavior, which typically occurs around days three to ten of a worker’s lifespan, is correlated with the largest HPG size. Previous work has shown that HPGs decrease in size as workers age, and are thus considered a marker for behavioral maturation [12]. Another study showed that precocious foragers, which are closer in age to nurse bees than to foragers, exhibit significantly smaller HPGs compared to normal, age-matched bees that are not yet foraging [43]. Our HPG size data further support the hypothesis that premature hive exiting behavior is a generalized stress response, given that the HPG size of prematurely exiting bees across all the stressors tested was significantly smaller when compared to that of their age-matched control counterparts, and statistically similar in size to their forager-aged control counterparts. Incidentally, we found that bees that prematurely exited the hive (which were two to seven days old) had HPGs that were the same size as those in healthy bees that were 18 to 25 days old.

HPG size is also influenced by nutrition, as higher protein consumption leads to increased HPG size [48]. However, we did not find any differences in weight between bees that performed the premature hive exiting behavior compared to those that did not. Therefore, we conclude that the background level of food available in all experimental colonies did not affect HPG size in the prematurely exiting bees. In the future, it would be interesting to test whether colonies faced with poor nutrition, particularly lack of pollen, could exhibit developing bees performing the behavior at higher rates compared to well-nourished colonies.

Following the evidence that exiting the hive prematurely is driven by general stress, we posit that this behavior could be a form of extremely accelerated age polyethism whereby the bees are so stressed that they leave the hive before they are physically able to fly, and therefore die outside the hive entrance soon thereafter. As previously mentioned, the size of the HPGs in all prematurely exiting bees was similar to the size of HPGs in forager-aged control counterparts, indicating that, in this physiological regard, premature hive exiting bees are similar to those that experience accelerated age polyethism [12]. Thus, general stress may not only cause precocious foraging behavior, but may also cause young bees that are unable to fly to prematurely exit the hive—a behavior that leads directly to death, and which has been previously unaccounted for in the literature. This behavior causes young bees to disappear before they are able to provide any service to the colony, ultimately depleting the workforce and wasting colony resources.

Perry et al. (2015) proposed that precocious foraging causes the symptoms of colony collapse disorder (CCD) [28]. Symptoms of collapsing colonies include the sudden loss of most of the worker population (except for a few remaining adult bees), abundant food resources still remaining, and the presence of the queen. We propose that the premature hive exiting behavior should be added to such models of colony dynamics, because high stress levels can cause a faster disappearance of young workers and/or a higher number of ineffective foragers. We further argue that this is an important behavior that could have major negative colony impacts, and one that speaks to the need of minimizing stress on honey bee colonies.

Future work regarding this novel behavior should include analyzing the expression of stress-related genes in prematurely exiting bees, particularly heat-shock proteins, as well as other physiological markers of precocious foraging, including expression levels of JH and Vg [29, 30, 49]. It would also be valuable to screen bees that prematurely exit the hive for the presence and abundance of honey bee-associated viruses, particularly Deformed wing virus (DWV), because high *Varroa* mite parasitization causes high background levels of DWV regardless of whether individual bees are parasitized during pupation [50]. Those additional data would be useful in parametrizing and calibrating a novel mathematical model that could be used to predict the threshold levels of developmental stress above which premature hive exiting behavior occurs at high enough rates that, when combined with other environmental stressors, could lead to colony collapse.

## Supplementary Information

Supplementary Material 1. Supplementary Figure 1. Mean and median distribution of the weight of honey bee workers that prematurely exited the hive, and those that did not perform the premature hive exiting behavior. The data were evaluated using a Wilcoxon rank sums test. A) Weight of bees from the cold stress trials. There was no significant difference in weight between bees that prematurely exited the hive and those that did not. B) Weight of bees from the *Varroa* mite parasitization stress trials. There was no significant difference in weight between bees that performed the premature hive exiting behavior and those that did not.

Supplementary Material 2. Supplementary Figure 2. Survivorship curves for honey bee workers that were stressed during pupation with either A) cold stress, B) heat stress, or C) *Varroa* mite parasitization. Statistical evaluation was done with a Kaplan Meier survivorship curve and a log rank test. Stressed beesdied significantly faster than bees in their respective control groupswhen they were exposed to cold stress, as well as to heat stress. There was a similar trend when bees were parasitized by *Varroa* mites, but not significantly so. Asterisksnext to the brackets represent statistically significant differences between treatment groups.

Supplementary Material 3. Supplementary Video 1. Two video examples of the premature hive exiting behavior in young honey bee workers. The bees that performed the behavior are circled prior to the start of each of the two videos. Each worker is individually tagged and is under five days of age. The focal bee in each of the videos can be seen walking on the runway of the hive’s entrance unattended and then falling off the end of the runway to the ground below.

## Data Availability

All data for this work are stored in the Texas Data Repository website and are available at https://doi.org/10.18738/T8/U5ND7E.
