# Supplementary material for: Stress drives premature hive exiting behavior that leads to death in young honey bee (Apis mellifera) workers
Source: Biol Res. 2024 Nov 27;57:92. doi: 10.1186/s40659-024-00569-z (PMC11600856; doi:10.1186/s40659-024-00569-z)
Supplement: Supplementary file 1 — Supplementary Material 1. Supplementary Figure 1. Mean and median distribution of the weight of honey bee workers that prematurely exited the hive, and those that did not perform the premature hive exiting behavior. The data were evaluated using a Wilcoxon rank sums test. A) Weight of bees from the cold stress trials. There was no significant difference in weight between bees that prematurely exited the hive and those that did not. B) Weight of bees from the Varroa mite parasitization stress trials. There was no significant difference in weight between bees that performed the premature hive exiting behavior and those that did not. [file 40659_2024_569_MOESM1_ESM.pptx]

## Slide 1
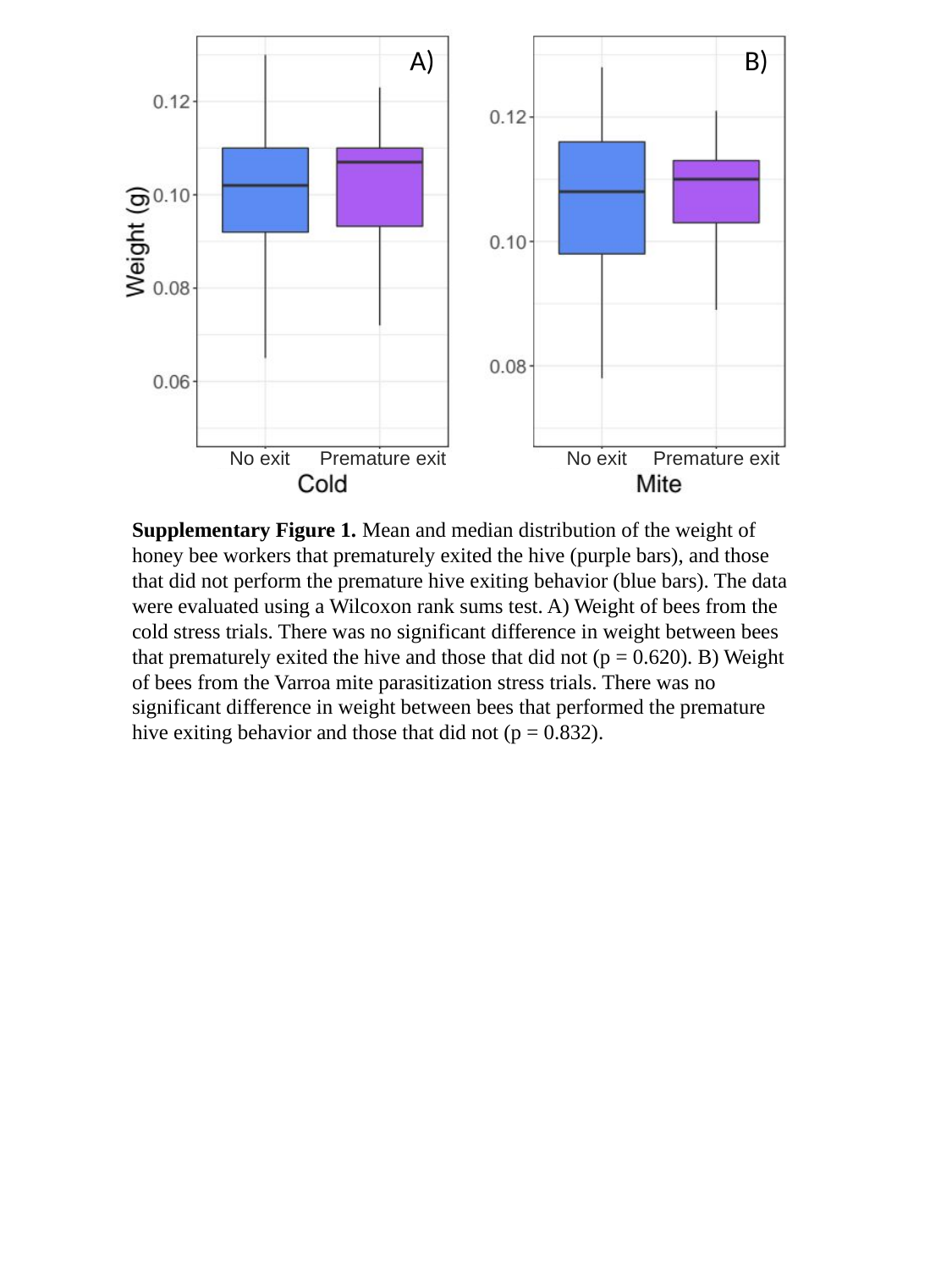

A)
B)
No exit
No exit
Premature exit
Premature exit
Supplementary Figure 1. Mean and median distribution of the weight of honey bee workers that prematurely exited the hive (purple bars), and those that did not perform the premature hive exiting behavior (blue bars). The data were evaluated using a Wilcoxon rank sums test. A) Weight of bees from the cold stress trials. There was no significant difference in weight between bees that prematurely exited the hive and those that did not (p = 0.620). B) Weight of bees from the Varroa mite parasitization stress trials. There was no significant difference in weight between bees that performed the premature hive exiting behavior and those that did not (p = 0.832).
