# Supplementary material for: Stress drives premature hive exiting behavior that leads to death in young honey bee (Apis mellifera) workers
Source: Biol Res. 2024 Nov 27;57:92. doi: 10.1186/s40659-024-00569-z (PMC11600856; doi:10.1186/s40659-024-00569-z)
Supplement: Supplementary file 2 — Supplementary Material 2. Supplementary Figure 2. Survivorship curves for honey bee workers that were stressed during pupation with either A) cold stress, B) heat stress, or C) Varroa mite parasitization. Statistical evaluation was done with a Kaplan Meier survivorship curve and a log rank test. Stressed beesdied significantly faster than bees in their respective control groupswhen they were exposed to cold stress, as well as to heat stress. There was a similar trend when bees were parasitized by Varroa mites, but not significantly so. Asterisksnext to the brackets represent statistically significant differences between treatment groups. [file 40659_2024_569_MOESM2_ESM.pptx]

## Slide 1
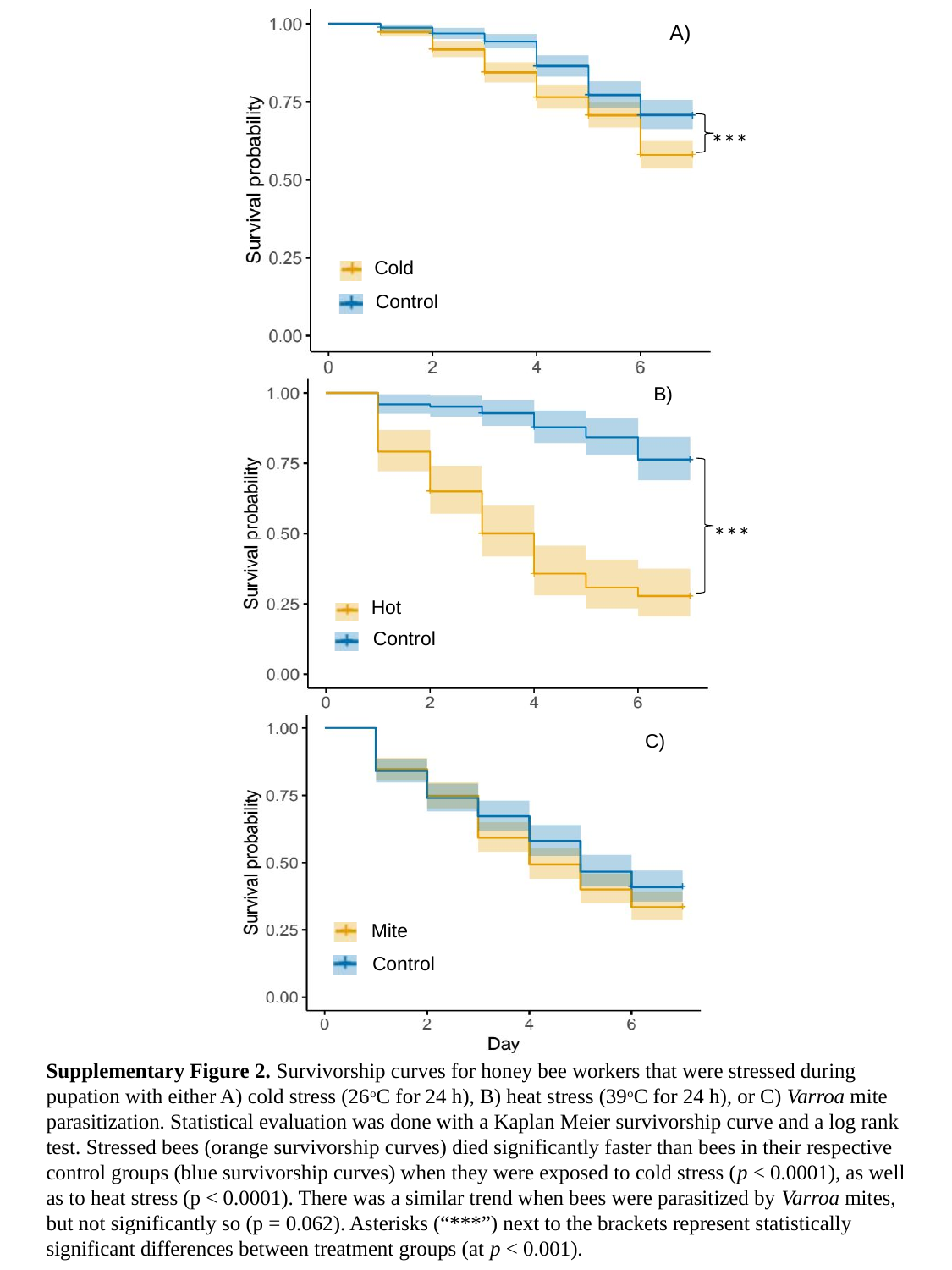

A)
***
Cold
Control
B)
***
Hot
Control
C)
Mite
Control
Supplementary Figure 2. Survivorship curves for honey bee workers that were stressed during pupation with either A) cold stress (26oC for 24 h), B) heat stress (39oC for 24 h), or C) Varroa mite parasitization. Statistical evaluation was done with a Kaplan Meier survivorship curve and a log rank test. Stressed bees (orange survivorship curves) died significantly faster than bees in their respective control groups (blue survivorship curves) when they were exposed to cold stress (p < 0.0001), as well as to heat stress (p < 0.0001). There was a similar trend when bees were parasitized by Varroa mites, but not significantly so (p = 0.062). Asterisks (“***”) next to the brackets represent statistically significant differences between treatment groups (at p < 0.001).
